# Observed Trough-Based Vancomycin Dosing Decisions Versus Model-Predicted Bayesian AUC-Guided Recommendations

**DOI:** 10.3390/healthcare14183127

**Published:** 2026-09-21

**Authors:** Wael A. Alghamdi

**Affiliations:** Department of Clinical Pharmacy, College of Pharmacy, King Khalid University, Abha 62223, Saudi Arabia; walghamdi@kku.edu.sa

**Keywords:** vancomycin, TDM, AUC, Bayesian dosing, dose optimization

## Abstract

**Background**: Vancomycin monitoring has traditionally relied on trough concentrations; however, recent guidelines recommend AUC-guided monitoring to optimize exposure while minimizing nephrotoxicity risk. This study compared trough-based clinical dosing decisions with predicted Bayesian AUC-guided recommendations. **Methods**: A retrospective study was conducted in adult patients receiving intravenous vancomycin between January and December 2025. Observed trough-based dose adjustments were compared with model-predicted Bayesian AUC-guided recommendations derived from steady-state trough concentrations. Agreement was evaluated using Cohen’s kappa and Bowker’s test. **Results**: Fifty-two patients were included. Agreement between trough-based and AUC-guided recommendations occurred in 26 cases (50%) with fair concordance (κ = 0.24). Among patients whose dose was maintained based on trough monitoring (*n* = 29), model-predicted AUC-guided recommendations indicated dose reduction in 48.3%. Among patients with acute kidney injury (*n* = 12), model-predicted AUC-guided recommendations suggested dose reduction in 58.3%, compared with 16.7% using trough-based decisions. **Conclusions**: Trough-based dosing showed limited agreement with model-predicted Bayesian AUC-guided recommendations, with frequent differences in the classification of vancomycin exposure and corresponding dosing recommendations.

## 1. Introduction

Vancomycin remains a cornerstone therapy for the treatment of serious Gram-positive infections. Therapeutic drug monitoring (TDM) has long been recommended to optimize vancomycin exposure while minimizing toxicity, particularly nephrotoxicity [1]. Historically, monitoring strategies relied on trough serum concentrations as a surrogate marker of drug exposure, with target trough concentrations of 15–20 mg/L recommended for severe infections.

However, accumulating pharmacokinetic and pharmacodynamic evidence indicates that vancomycin efficacy is best described by the area under the concentration–time curve to minimum inhibitory concentration ratio (AUC/MIC), while trough concentrations correlate inconsistently with this exposure parameter [1]. In addition, higher trough concentrations have been associated with an increased risk of nephrotoxicity without consistent improvement in clinical outcomes [1,2]. These observations have raised concerns regarding the reliability of trough concentrations as a sole surrogate for vancomycin exposure.

In response to these findings, revised consensus guidelines for therapeutic monitoring of vancomycin were published in 2020. These guidelines recommend targeting an AUC/MIC ratio of 400–600 (assuming an MIC of 1 mg/L) rather than relying solely on trough concentrations for dose adjustment, with the goal of optimizing antimicrobial efficacy while reducing the risk of nephrotoxicity [1]. Despite these recommendations, trough-based monitoring remains widely used in many clinical settings because of its relative simplicity and familiarity. Implementation of AUC-guided monitoring remains variable, with barriers including limited familiarity with AUC methods, software requirements, and difficulties related to concentration sampling and documentation [3,4,5]. In routine retrospective practice, application of AUC-guided monitoring is often limited by the availability of only a single steady-state trough concentration rather than paired concentrations. Although one-sample Bayesian estimation is less precise than approaches based on richer sampling, it can still provide individualized exposure estimates by integrating observed concentrations with prior population pharmacokinetic information [1,6,7].

Consequently, discrepancies may occur between dosing decisions based on trough concentrations and those that would be recommended using AUC-guided monitoring. Evaluating the extent and pattern of such discordance in real-world clinical practice may help clarify how trough-based clinical dosing decisions differ from those derived from AUC-based assessment. Therefore, the primary objective was to evaluate concordance between observed trough-based clinical dosing decisions and model-predicted Bayesian AUC-guided recommendations. In addition, the study explored the relationship between vancomycin exposure parameters and the occurrence of acute kidney injury.

## 2. Materials and Methods

### 2.1. Study Design and Population

A retrospective observational study was conducted at a secondary care hospital in the Aseer region, Saudi Arabia. The study utilized electronic health records of adult patients who received intravenous vancomycin between January and December 2025. Ethical approval for this study was granted by the Aseer Institutional Review Board (approval number: F10-2-2025). All patient data were anonymized prior to analysis to ensure confidentiality.

Adult patients aged ≥18 years who received intravenous vancomycin during the study period were screened for eligibility based on the availability of complete required data and a documented vancomycin trough concentration. Patients were included if at least one steady-state vancomycin trough concentration, obtained after at least three administered doses, was available for TDM. When multiple TDM measurements were available, only the first trough concentration used for clinical dose adjustment was included in the analysis. Patients were excluded if they were receiving dialysis, received fewer than three doses of vancomycin, had vancomycin concentrations that did not represent true trough concentrations (i.e., not obtained within 1 h before the next scheduled dose), had undocumented medication administration times that precluded confirmation of trough status, or had the first eligible TDM obtained more than 7 days after vancomycin initiation.

### 2.2. Data Collection

Patient demographic and baseline clinical characteristics, including age, sex, weight, height, body mass index, serum creatinine, and creatinine clearance, were collected. Vancomycin treatment–related variables were also recorded, including loading dose (when administered), maintenance dose, dosing frequency, daily dose, and TDM concentrations. Serum vancomycin concentrations were quantified using the Emit^®^ 2000 Vancomycin Assay, a homogeneous enzyme immunoassay, on a Beckman Coulter AU700 clinical chemistry analyzer (Beckman Coulter, Inc., Brea, CA, USA). Creatinine clearance was calculated using the Cockcroft–Gault equation.

### 2.3. Vancomycin Exposure Assessment and Outcomes

Vancomycin exposure was assessed using both trough-based monitoring and predicted AUC. For each patient, the measured steady-state trough concentration was used to estimate AUC using VancoCalc, a freely accessible, open-source Bayesian vancomycin dosing platform developed for adult patients and evaluated in hospitalized populations [8]. The platform supports AUC estimation from a single measured concentration, with model selection incorporating patient characteristics including obesity and critical illness status. VancoCalc applies published one-compartment population pharmacokinetic models according to patient characteristics, including general noncritically ill patients, critically ill patients, extremely obese patients, and obese critically ill patients [8]. Obesity was defined as BMI ≥ 30 kg/m^2^, and critical illness was operationalized as ICU admission at the time of the evaluated TDM episode. Patient-specific clinical and concentration data are then incorporated through Bayesian estimation to derive AUC. A single steady-state trough concentration was used because this reflected the TDM data available in routine clinical practice. Although Bayesian AUC estimation can be performed using a single concentration, estimates based on two appropriately timed concentrations may provide greater precision, particularly in patients with altered pharmacokinetics. In the present study, patient age, sex, weight, height, serum creatinine, critical illness status, vancomycin dosing history, trough concentration, and sampling time were entered into the calculator to estimate AUC. Estimated AUC values were interpreted according to the 2020 consensus guidelines for vancomycin therapeutic monitoring, which recommend a target AUC range of 400–600 mg·h/L [1].

The primary outcome was the agreement between observed trough-based clinical dose adjustments and predicted AUC-guided dosing recommendations. Observed trough-based dose adjustment categories were assigned according to the actual documented clinical decision following trough measurement and were classified as increase dose, maintain dose, or decrease dose. The observed trough-based dosing decisions reflected routine clinical practice and were not determined solely by predefined trough thresholds. Predicted AUC-guided recommendations were categorized according to the 2020 consensus guideline target range, with estimated AUC < 400 mg·h/L classified as increase dose, AUC 400–600 mg·h/L as maintain dose, and AUC > 600 mg·h/L as decrease dose. Predicted AUC-guided recommendation categories were assigned using the predefined objective cutoffs described above. Because isolate-specific MIC data were not consistently available, an MIC of 1 mg/L was assumed when interpreting the guideline-recommended AUC/MIC target. Accordingly, an AUC of 400–600 mg·h/L was considered the target exposure range. The secondary outcome was acute kidney injury (AKI) occurring during vancomycin therapy. AKI was defined as an increase in serum creatinine of ≥0.3 mg/dL within 48 h or a ≥50% increase from baseline within 7 days, according to the KDIGO criteria and the 2020 revised consensus guideline for vancomycin therapeutic monitoring [1,9].

### 2.4. Statistical Analysis

Descriptive statistics were used to summarize baseline patient characteristics and treatment variables. Continuous variables were reported as median and interquartile range (IQR), while categorical variables were reported as frequencies and percentages. Agreement between trough-based clinical dose adjustments and predicted AUC-guided dosing recommendations was evaluated using Cohen’s kappa (κ) statistic with corresponding 95% confidence intervals. Bowker’s test of symmetry was performed to assess the pattern of discordance between the two dosing strategies. AKI incidence in patients with AUC > 600 mg·h/L versus ≤600 mg·h/L was compared using Fisher’s exact test, with the risk ratio (RR) and 95% confidence interval reported as an effect estimate. A two-sided *p*-value < 0.05 was considered statistically significant. Statistical analyses were performed using JMP^®^ v18 (SAS Institute, Cary, NC, USA).

## 3. Results

### 3.1. Patient Characteristics

A total of 52 patients were included in the analysis. Of the 96 patients screened for eligibility, 44 were excluded because of dialysis (*n* = 2), fewer than 3 administered vancomycin doses before the first eligible TDM measurement (*n* = 17), non-trough concentrations (*n* = 9), undocumented medication administration times that precluded confirmation of trough status (*n* = 9), or delayed TDM obtained more than 7 days after vancomycin initiation (*n* = 7). Baseline demographic and clinical characteristics are summarized in Table 1. The median age was 41.0 years (IQR, 27.8–61.2), and 37 patients (71.2%) were male. The median creatinine clearance was 70.9 mL/min (IQR, 61.0–95.2), the median vancomycin daily dose was 30.8 mg/kg/day (IQR, 28.6–38.1), and the median steady-state trough concentration was 10.9 mg/L (IQR, 7.6–15.3). Acute kidney injury occurred in 12 patients (23.1%).

### 3.2. Comparison of Trough-Based and AUC-Guided Dose Adjustments

Overall agreement between observed trough-based clinical dosing decisions and model-predicted AUC-guided recommendations occurred in 26 of 52 patients (50.0%), with discordant recommendations in the remaining 26 patients (50.0%). Cohen’s kappa was 0.24 (95% CI 0.05–0.43; *p* = 0.002), indicating fair agreement. Bowker’s test of symmetry demonstrated significant directional discordance between the two approaches (χ^2^ = 22.4, *p* < 0.001). The most frequent discordant pattern was a trough-based decision to maintain the dose with an AUC-guided recommendation to decrease it, occurring in 14 of 29 patients (48.3%) in the maintain-dose group. Two patients had opposite-direction recommendations, with a trough-based dose increase versus an AUC-guided dose decrease.

**Figure 1:**
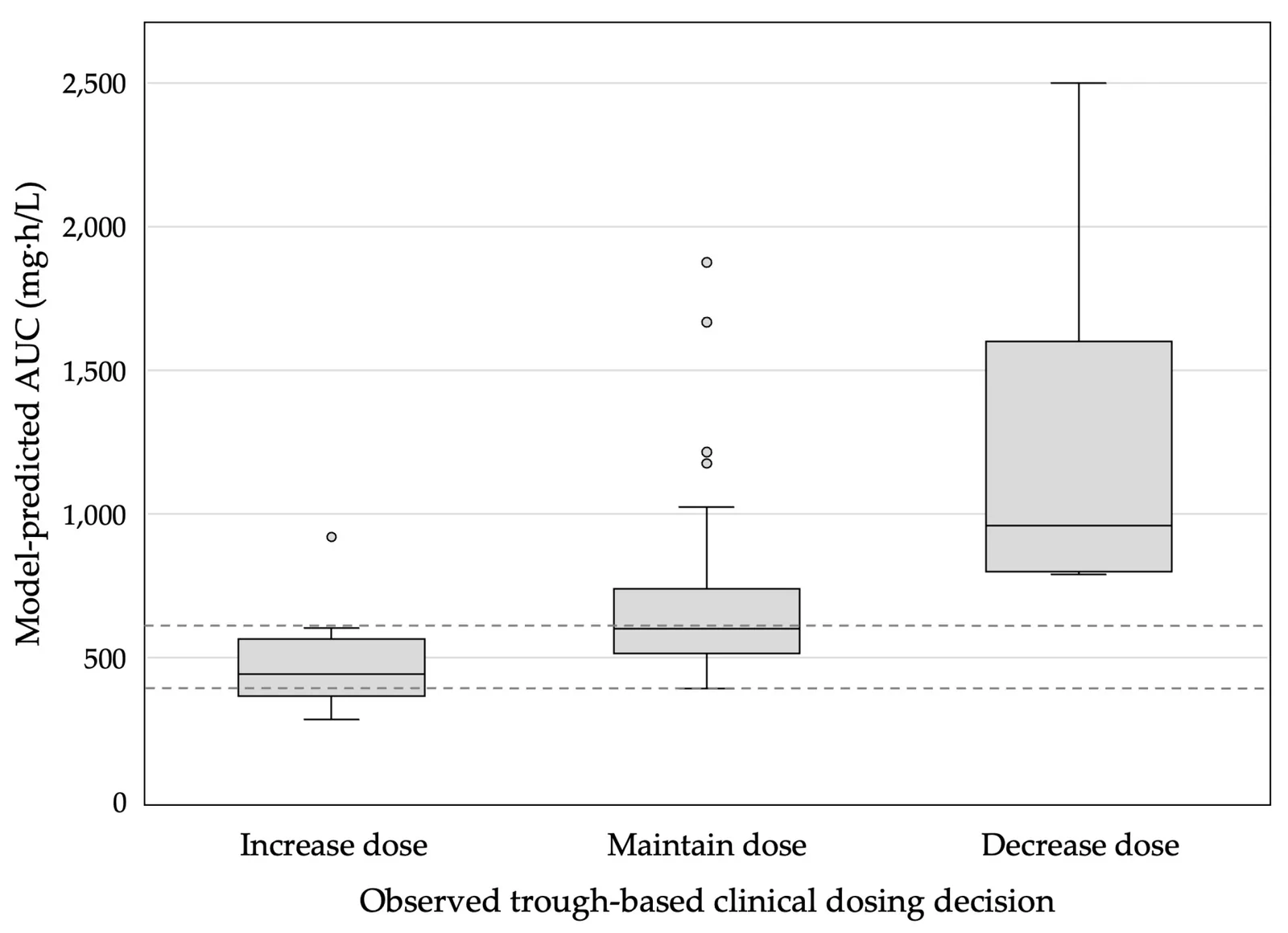
Distribution of model-predicted Bayesian AUC according to observed trough-based clinical dosing decisions. Horizontal dashed lines indicate the recommended AUC target range of 400–600 mg·h/L.

The distribution of dosing recommendations is shown in Table 2. Among the 16 patients for whom the observed trough-based decision was to increase the dose, the AUC-guided recommendation was to increase the dose in 5 (31.3%), maintain the dose in 9 (56.3%), and decrease the dose in 2 (12.5%). Among the 29 patients for whom the observed decision was to maintain the dose, the AUC-guided recommendation was to increase the dose in 1 (3.4%), maintain the dose in 14 (48.3%), and decrease the dose in 14 (48.3%). Among the 14 patients with a trough-based maintain decision and an AUC-guided decrease recommendation, the median trough concentration was 13.7 mg/L (IQR, 10.1–26.0; range 5.6–42.7). Trough concentrations were <10 mg/L in 4 patients (28.6%), 10–15 mg/L in 4 (28.6%), 15–20 mg/L in 2 (14.3%), and >20 mg/L in 4 (28.6%). All 7 patients for whom the observed trough-based decision was to decrease the dose also had an AUC-guided recommendation to decrease the dose. In this subgroup, the median trough concentration was 23.8 mg/L (IQR, 21.5–29.2), and the median predicted AUC was 960 mg·h/L (IQR, 854.5–1355.5); six of seven patients (85.7%) had trough concentrations > 20 mg/L, and all had predicted AUC > 600 mg·h/L. The distribution of model-predicted AUC values according to the observed trough-based clinical dosing decision is shown in Figure 1.

### 3.3. Acute Kidney Injury and Vancomycin Exposure

The distribution of dosing recommendations among patients who developed acute kidney injury is shown in Table 3. Among the 12 patients with AKI, model-predicted AUC-guided recommendations suggested dose reduction in 7 patients (58.3%) and dose maintenance in 5 patients (41.7%), with no recommendation for dose increase. In contrast, trough-based clinical decisions in the same subgroup were to increase the dose in 4 patients (33.3%), maintain the dose in 6 patients (50.0%), and decrease the dose in 2 patients (16.7%).

The incidence of AKI according to vancomycin exposure parameters is presented in Table 4. Based on AUC categories, AKI occurred in 0 (0.0%) with AUC < 400 mg·h/L, 2 (22.2%) with AUC 400–500 mg·h/L, 3 (21.4%) with AUC 500–600 mg·h/L, and 7 (30.4%) with AUC > 600 mg·h/L. Based on trough concentration categories, AKI occurred in 5 (22.7%) with trough concentrations < 10 mg/L, 3 (20.0%) with trough concentrations of 10–15 mg/L, 1 (20.0%) with trough concentrations of 15–20 mg/L, and 3 (30.0%) with trough concentrations > 20 mg/L. Among the eight patients who developed AKI with trough concentrations < 15 mg/L, three (37.5%) had an estimated AUC > 600 mg·h/L, whereas five (62.5%) had an estimated AUC within the 400–600 mg·h/L target range. When AUC > 600 mg·h/L was compared with AUC ≤ 600 mg·h/L, AKI occurred in 7 of 23 (30.4%) versus 5 of 29 patients (17.2%), respectively (RR 1.77, 95% CI 0.64–4.84; *p* = 0.329).

## 4. Discussion

In this single-center retrospective study, trough-based clinical dose adjustments showed limited agreement with predicted AUC-guided recommendations. Overall agreement was observed in only half of the cohort, with fair concordance by Cohen’s kappa and significant asymmetry by Bowker’s test, indicating that the two monitoring approaches frequently led to different dosing decisions. The most clinically relevant discordance was observed among patients whose dose was maintained based on trough monitoring, nearly half of whom would have been recommended for dose reduction using AUC-guided assessment. Among patients who developed AKI, model-predicted AUC-guided recommendations favored dose reduction more often than trough-based decisions. In addition, AKI incidence was numerically highest in patients with AUC values > 600 mg·h/L, although the difference compared with AUC ≤ 600 mg·h/L was not statistically significant.

These findings are consistent with the rationale underlying the 2020 revised consensus guideline for vancomycin therapeutic monitoring, which recommends targeting an AUC/MIC of 400–600 rather than relying on trough-only monitoring, with the goal of balancing efficacy and safety [1]. The present findings also align with emerging literature showing that trough concentrations and AUC values are frequently discordant. In a retrospective evaluation by Chen et al., 75% of trough/AUC pairs were discordant, and 69% of troughs between 15 and 20 mg/L were associated with an AUC > 600 mg·h/L [6]. More recently, Johnson-Louis et al. reported discordance in 52.9% of vancomycin trough/AUC pairs in patients at high risk of nephrotoxicity, with 57% of troughs within the 15–20 mg/L range corresponding to an AUC > 600 mg·h/L [10]. The 50% discordance observed in the present study is therefore directionally consistent with prior reports and supports the concern that trough-based monitoring may not reliably reflect total vancomycin exposure. In a Saudi cohort, Bayesian AUC assessment identified target exposure in only 37.1% of patients despite 54% having trough concentrations within the conventional target range, and trough concentrations of 15–20 mg/L were strongly associated with AUC values > 600 mg·h/L [11].

The pattern observed in Table 2 is particularly relevant clinically. Although trough-based monitoring led to dose maintenance in 29 patients, AUC-guided assessment recommended dose reduction in 14 of those cases. This indicates that a substantial subset of patients whose doses were maintained based on trough monitoring were classified by Bayesian AUC estimation as having exposure above the recommended therapeutic window. Conversely, agreement was complete among patients with trough-based dose reductions; six of seven had trough concentrations > 20 mg/L, and all had predicted AUC values > 600 mg·h/L, suggesting that markedly elevated trough concentrations may still identify excessive vancomycin exposure. Together, these findings are consistent with the 2020 guideline’s shift away from trough-only targets and toward AUC-based monitoring [1].

Bayesian AUC estimation can use limited concentration sampling by integrating observed concentrations with prior population pharmacokinetic information [8,12]. However, the precision of the estimate depends on the sampling strategy and underlying population model. In a retrospective study of 978 hospitalized adults, one-concentration Bayesian estimates showed 88.5% clinical agreement with two-concentration Bayesian estimates, although greater variability was observed with single-concentration estimation [7]. Furthermore, different Bayesian population models may produce clinically meaningful differences in estimated AUC and resulting dose classifications, particularly in critically ill patients [13]. These findings support the practical feasibility of limited-sampling Bayesian estimation while emphasizing the uncertainty inherent in single-concentration approaches.

The AKI findings provide additional context regarding the observed dosing discordance. In the AKI subgroup, model-predicted AUC-guided recommendations suggested dose reduction in 58.3% of cases, whereas trough-based decisions recommended dose reduction in only 16.7%. This does not establish that AKI would have been prevented had AUC-guided monitoring been used prospectively; rather, it demonstrates that the model-predicted AUC assessment classified exposure differently from the observed trough-based clinical decisions in several patients who developed AKI. This interpretation is supported by larger secondary studies and meta-analyses. Zasowski et al. reported that the predictive performance of vancomycin exposure for nephrotoxicity was maximized at daily AUC values between 600 and 800 mg·h/L, supporting the clinical relevance of exposure thresholds above the currently recommended target range [2]. In addition, observational implementation studies and meta-analyses have generally reported lower nephrotoxicity with AUC-guided monitoring than with trough-guided monitoring [14,15,16,17,18].

Abdelmessih et al. reported that AUC-guided dosing strategies were associated with significantly less vancomycin-induced AKI than trough-guided strategies in a meta-analysis of 10 studies including 4231 patients [14]. Similarly, Lim et al. found a lower pooled prevalence of nephrotoxicity with AUC-guided TDM than with trough-guided monitoring, with a lower overall odds of nephrotoxicity in the AUC-guided group [15].

In our cohort, AKI incidence was numerically highest in the group with AUC > 600 mg·h/L. This pattern is directionally consistent with prior literature and with the current recommended upper exposure boundary of 600 mg·h/L [1,2]. Notably, eight of the 12 AKI events occurred in patients with trough concentrations < 15 mg/L. Of these, three had estimated AUC values > 600 mg·h/L, whereas five remained within the recommended AUC range. This finding indicates that AKI occurred across both trough and AUC exposure categories and, given the small number of events and unavailable clinical risk factors, should not be attributed to vancomycin exposure alone. AKI was identified according to KDIGO criteria during vancomycin therapy but was not formally adjudicated as being attributable to vancomycin; therefore, other clinical causes of renal injury could not be excluded. A similar distinction between trough concentration and estimated total exposure has been observed in studies specifically examining nephrotoxicity. In one retrospective analysis, an AUC > 600 mg·h/L estimated from a single trough concentration was independently associated with AKI, whereas a trough concentration ≥15 mg/L was not [19]. The relationship between vancomycin exposure and AKI should be interpreted cautiously. Although higher vancomycin exposure has been associated with nephrotoxicity in previous studies, the temporal relationship cannot be established from the present retrospective data. Declining renal function may itself reduce vancomycin clearance and result in higher trough concentrations and AUC estimates. Therefore, the observed association between higher exposure and AKI should not be interpreted as evidence that elevated vancomycin exposure necessarily preceded or caused the renal injury.

This study has several strengths. It evaluated actual clinical trough-based dose adjustments and directly compared them with predicted AUC-guided recommendations, addressing a pragmatic and clinically relevant question. In addition, the analysis incorporated both dosing decision discordance and nephrotoxicity patterns, allowing a broader assessment of the potential clinical implications of the observed discordance between the two monitoring approaches. The use of standardized AKI criteria and clearly defined dosing categories further facilitated clinically interpretable comparisons. However, several limitations should be acknowledged. The single-center design and small cohort limit the generalizability of the findings, particularly to critically ill patients, who represented only 11.5% of the cohort, and to patients with severe MRSA infection or substantially altered renal clearance. The retrospective design limits causal inference, and only 12 patients developed AKI, which precluded reliable multivariable adjustment for potential confounders because of the limited number of events. In addition, AUC estimates were derived from a single steady-state trough concentration rather than two concentrations, which may have reduced the precision of Bayesian exposure estimates in some patients. Furthermore, only the first eligible TDM episode per patient was analyzed. Excluding patients whose first eligible TDM occurred more than 7 days after vancomycin initiation may also have introduced selection bias by preferentially including patients monitored earlier in therapy. Other clinically relevant contributors to nephrotoxicity, including baseline comorbidities, concomitant nephrotoxic medications, hemodynamic instability, infection severity, duration of vancomycin therapy, and other AKI risk factors, were not consistently available for analysis. Accordingly, the AKI findings should be interpreted as exploratory.

## 5. Conclusions

This retrospective concordance analysis demonstrated only fair agreement between trough-based vancomycin dosing decisions and model-predicted Bayesian AUC-guided recommendations, with clinically meaningful discordance occurring frequently. Prospective studies are warranted to determine whether implementing AUC-guided monitoring improves vancomycin safety and dosing optimization. Prospective studies are warranted to determine whether implementing AUC-guided monitoring improves vancomycin safety and dosing optimization.

## Figures and Tables

**Table 1:** Baseline demographic and clinical characteristics of patients receiving vancomycin.

Characteristic	Value
Sex, *n* (%)	
Male	37 (71.2)
Female	15 (28.8)
Age, years	41.0 (27.8–61.2)
Weight, kg	70.0 (65.0–75.5)
Height, cm	167.5 (160.0–170.0)
Body mass index, kg/m^2^	24.2 (24.2–27.4)
Obesity, *n* (%)	9 (17.3)
ICU admission, *n* (%)	6 (11.5)
Creatinine clearance, mL/min	70.9 (61.0–95.2)
Serum creatinine, mg/dL	1.2 (0.8–1.5)
Vancomycin daily dose, mg/kg/day	30.8 (28.6–38.1)
Vancomycin trough concentration, mg/L	10.9 (7.6–15.3)
Acute kidney injury, *n* (%)	12 (23.1)

Data are presented as median (IQR) unless otherwise indicated.

**Table 2:** Comparison of observed trough-based clinical dosing decisions and predicted Bayesian AUC-guided dosing recommendations.

Observed Clinical Adjustment (Trough-Based)	Predicted AUC-Guided Recommendation			Total
	Increase Dose	Maintain Dose	Decrease Dose	
Increase dose	5 (31.3%)	9 (56.3%)	2 (12.5%)	16
Maintain dose	1 (3.4%)	14 (48.3%)	14 (48.3%)	29
Decrease dose	0 (0.0%)	0 (0.0%)	7 (100%)	7
Total	6	23	23	52

Values are presented as *n* (%). Percentages represent row percentages.

**Table 3:** Comparison of dosing recommendations among patients who developed acute kidney injury.

Dosing Recommendation	AKI Cases with AUC-Guided Recommendation *n* (%)	AKI Cases with Trough-Based Recommendation *n* (%)
Increase dose	0 (0.0%)	4 (33.3%)
Maintain dose	5 (41.7%)	6 (50.0%)
Decrease dose	7 (58.3%)	2 (16.7%)

**Table 4:** Incidence of acute kidney injury according to vancomycin exposure parameters.

Exposure Parameter	Number of Patients	AKI Cases, *n* (%)
AUC (mg·h/L)		
<400	6	0 (0.0%)
400–500	9	2 (22.2%)
500–600	14	3 (21.4%)
>600	23	7 (30.4%)
Trough concentration (mg/L)		
<10	22	5 (22.7%)
10–15	15	3 (20.0%)
15–20	5	1 (20.0%)
>20	10	3 (30.0%)

## Data Availability

The data used in this study are not publicly available due to institutional and ethical restrictions.
